# Genetic variants associated with inherited cardiovascular disorders among 13,131 asymptomatic older adults of European descent

**DOI:** 10.1038/s41525-021-00211-x

**Published:** 2021-06-16

**Authors:** Paul Lacaze, Robert Sebra, Moeen Riaz, Jodie Ingles, Jane Tiller, Bryony A. Thompson, Paul A. James, Diane Fatkin, Christopher Semsarian, Christopher M. Reid, Andrew M. Tonkin, Ingrid Winship, Eric Schadt, John J. McNeil

**Affiliations:** 1 Department of Epidemiology and Preventive Medicine, School of Public Health and Preventive Medicine, Monash University, Melbourne, VIC Australia; 2 Department of Genetics and Genomic Sciences, Icahn Institute for Data Science and Genomic Technology, Icahn School of Medicine at Mount Sinai, New York, NY USA; 3 Sema4, a Mount Sinai venture, Stamford, CT USA; 4 Cardio Genomics Program at Centenary Institute and Faculty of Medicine and Health, The University of Sydney, Camperdown, NSW Australia; 5 Genomic Medicine and Family Cancer Clinic, Royal Melbourne Hospital, Parkville, VIC Australia; 6 Department of Genomic Medicine, Family Cancer Clinic, Department of Medicine, Department of Pathology, Royal Melbourne Hospital, University of Melbourne, Parkville, VIC Australia; 7 Molecular Cardiology and Biophysics Division, Victor Chang Cardiac Research Institute, Darlinghurst, NSW Australia; 8 St Vincent’s Clinical School, Faculty of Medicine, University of New South Wwales Sydney, Kensington, NSW Australia; 9 Cardiology Department, St. Vincent’s Hospital, Darlinghurst, NSW Australia; 10 Agnes Ginges Centre for Molecular Cardiology at Centenary Institute and Faculty of Medicine and Health, The University of Sydney, New South Wales, Australia; 11 Department of Cardiology, Royal Prince Alfred Hospital, Sydney, New South Wales, Australia; 12 School of Public Health, Curtin University, Perth, WA Australia

**Keywords:** Medical genomics, Genetic variation

## Abstract

Genetic testing is used to optimise the management of inherited cardiovascular disorders that can cause sudden cardiac death. Yet more genotype–phenotype correlation studies from populations not ascertained on clinical symptoms or family history of disease are required to improve understanding of gene penetrance. We performed targeted sequencing of 25 genes used routinely in clinical genetic testing for inherited cardiovascular disorders in a population of 13,131 asymptomatic older individuals (mean age 75 years) enrolled in the ASPREE trial. Participants had no prior history of cardiovascular disease events, dementia or physical disability at enrolment. Variants were classified following ACMG/AMP standards. Sudden and rapid cardiac deaths were clinically adjudicated as ASPREE trial endpoints, and assessed during mean 4.7 years of follow-up. In total, 119 participants had pathogenic/deleterious variants in one of the 25 genes analysed (carrier rate of 1 in 110 or 0.9%). Participants carried variants associated with hypertrophic cardiomyopathy (*N* = 24), dilated cardiomyopathy (N = 29), arrhythmogenic right-ventricular cardiomyopathy (*N* = 22), catecholaminergic polymorphic ventricular tachycardia (*N* = 4), aortopathies (*N* = 1), and long-QT syndrome (*N* = 39). Among 119 carriers, two died from presumed sudden/rapid cardiac deaths during follow-up (1.7%); both with pathogenic variants in long-QT syndrome genes (*KCNQ1*, *SCN5A*). Among non-carriers, the rate of sudden/rapid cardiac deaths was significantly lower (0.08%, 11/12936, *p* < 0.001). Variants associated with inherited cardiovascular disorders are found in asymptomatic individuals aged 70 years and older without a history of cardiovascular disease.

## Introduction

Genetic testing is part of the clinical management of inherited cardiovascular disorders, including conditions that can cause sudden cardiac death, such as hypertrophic, dilated and arrhythmogenic cardiomyopathies, aortopathies, and inherited arrhythmia syndromes^1,2^. However, incomplete penetrance and variable expressivity of genes are hallmarks of these conditions, complicating the interpretation of genetic testing results^3^. Many genes currently used in genetic testing panels for inherited cardiovascular disorders and sudden cardiac death are not supported by robust gene-disease association or accurate gene penetrance estimates^4–6^. As a result, there is a risk of misclassification of variants detected in these genes, especially when detected in the absence of clinical symptoms or a family history of related disease.

Most clinical studies for inherited cardiovascular disorder genes, to date, have involved patients and families affected by arrhythmias and sudden cardiac death^7,8^. This has created an ascertainment bias towards clinically affected individuals and family members, which in turn, has likely influenced gene penetrance estimates for disease-associated genes. Recently, studies from population-based cohorts not ascertained on clinical symptoms or criteria have been published^9–15^. These studies have begun to provide a less biased estimate of gene penetrance and the clinical effects of germline cardiovascular disorder gene variants. However, many studies have involved cross-sectional analysis and require longitudinal follow-up to calculate lifetime risk. Clinical studies from populations not ascertained on related clinical symptoms or family history of cardiovascular disease are required to understand gene penetrance.

Here, we report the prevalence and clinical impact of pathogenic variants identified in genes used routinely in clinical genetic testing for inherited cardiovascular disorders, among a population of 13,131 asymptomatic individuals aged 70 years and older without a history of atherothrombotic cardiovascular disease events. These individuals were enrolled in the placebo-controlled ASPirin in Reducing Events in the Elderly (ASPREE) trial^16–18^. We examine the clinical effect of the variants detected using adjudicated sudden and rapid cardiac deaths in the trial, to average age 79 years. Our study provides an important opportunity to assess the prevalence and clinical effect of variants associated with inherited cardiovascular disorders, in a population not ascertained by clinical features related to the conditions in question.

## Results

### Prevalence of variants associated with inherited cardiovascular disorders

The 13,131 sequenced ASPREE participants were mostly white/Caucasian by self-reported ethnicity, 54% female, with low rates of obesity (28%, BMI ≥ 30 kg/m^2^) (Table 1). After DNA sequencing and variant curation^19^, we detected 119 individuals in the ASPREE population with pathogenic or likely pathogenic variants in genes associated with inherited cardiovascular disorders (including 25 individuals with deleterious or likely deleterious *TTN*-truncating variants) (Table 2). Overall, this corresponded to a carrier rate of 0.9% or 1 in 110 ASPREE participants for the 25 genes analysed.

**Table 1:** Baseline characteristics of sequenced participants.

	ASPREE
Total population size	13,131
Female sex—no. (%)	7056 (54)
Age at enrolment in years (mean)	75
Age after follow-up in years (mean)	79
Age range in years—no. (%)	
70–74 years	7894 (60)
75–79 years	3406 (26)
≥80 years	1831 (14)
Race or ethnic group^a^—no. (%)	
White/Caucasian	12,953 (99)
Other	178 (1)
Obese (BMI ≥ 30 kg/m^2^)^b^ (%)	28
Current smoking (%)	4
Heart, stroke or vascular disease^c^ (%)	0

Participants were enrolled in the ASPREE trial, aged 70 years and older, without a prior history or current diagnosis of atherothrombotic cardiovascular disease, dementia diagnosis, physical disability or life-threating cancer diagnoses.

^a^Self-report.

^b^Obese was defined as body-mass index (weight in kilograms divided by the square of the height in meters) of ≥30.

^c^Defined by the ASPREE (ASPirin in Reducing Events in the Elderly) Study Protocol.

**Table 2:** Summary of variant prevalence.

Participants of the ASPREE study (*N* = 13,131)	Genes	Variants	Carriers	Carrier rate	Carrier %
Condition *Associated genes*					
Hypertrophic cardiomyopathy * MYBPC3, MYH7, TNNI3, TNNT2, TPM1, ACTC1, MYL2, MYL3, GLA*	9	20	24	1 in 547	0.2%
Dilated cardiomyopathy * TTNtv, LMNA, RBM20, PLN, BAG3, FLNC*	6	26	29	1 in 453	0.2%
Arrhythmogenic right-ventricular cardiomyopathy * PKP2, DSP, DSC2, DSG2, TMEM43*	5	14	22	1 in 597	0.2%
Catecholaminergic polymorphic ventricular tachycardia * RYR2*	1	4	4	1 in 3283	0.03%
Marfan syndrome and familial thoracic aortic aneurysms and dissections *FBN1*	1	1	1	1 in 13131	0.007%
Familial long-QT syndrome types 1/2/3 * KCNQ1, KCNH2, SCN5A*	3	24	39	1 in 337	0.3%
Total	25	89	119	1 in 110	0.9%

We performed targeted sequencing of 25 genes used routinely in clinical genetic testing for inherited cardiovascular disorders in 13,131 asymptomatic individuals aged 70 or older (mean age 75 years) enrolled into the ASPREE trial. At the time of enrolment, participants had no prior history or symptoms of atherothrombotic cardiovascular disease, dementia or physical disability. Variants were classified as pathogenic/likely pathogenic following ACMG/AMP standards or deleterious/likely deleterious for *TTN-*truncating variants (TTNtv).

We detected 89 different variants meeting our inclusion criteria in the 25 disease-associated genes (Table 3). For detailed information on the 89 variants detected, including genomic coordinates, clinical annotations, supporting evidence of pathogenicity, and population allele frequencies from different reference populations, see Supplementary Data 1. The majority of variants were found as singletons (in only one participant). However, some variants were detected in up to seven different participants in the population. These included the *KCNQ1* NM_000218.3:c.1552 C > T, p.(Arg518Ter) Swedish founder mutation.; associated with a milder long-QT syndrome phenotype^20^, and the *PKP2* NM_004572.3:c.2146-1 G > C variant; associated with incomplete segregation in families affect by arrhythmogenic right-ventricular cardiomyopathy^21^. We performed validation by Sanger sequencing on a random selection of 10 of the 89 variants (11%) and found 100% technical concordance.

**Table 3:** Pathogenic or deleterious variants in genes associated with inherited cardiovascular disorders among 13,131 asymptomatic individuals aged 70 years and older without a history of cardiovascular events.

Gene	Variant	rsID	Allele Count	Call	gnomAD-NFE MAF v2.1.1
Hypertrophic cardiomyopathy (*MYBPC3, MYH7, TNNI3, TNNT2, TPM1, ACTC1, MYL2, MYL3, GLA*), *N* = 24 carriers					
* MYBPC3*	NM_000256.3(MYBPC3):c.3627 + 1 G > A	rs397516031	1	Pathogenic	0
* MYBPC3*	NM_000256.3(MYBPC3):c.2864_2865delCT, p.(Pro955Argfs)	rs397515990	1	Pathogenic	0
* MYBPC3*	NM_000256.3(MYBPC3):c.2558delG, p.(Gly853Alafs)	rs397515977	1	Pathogenic	0
* MYBPC3*	NM_000256.3(MYBPC3):c.2459 G > A, p.(Arg820Gln)	rs2856655	1	Pathogenic	0.00003541
* MYBPC3*	NM_000256.3(MYBPC3):c.2373dupG, p.(Trp792ValfsTer41)	rs187830361	1	Pathogenic	0.00004277
* MYBPC3*	NM_000256.3(MYBPC3):c.2374 T > C, p.(Trp792Arg)	rs187830361	1	Likely pathogenic	0
* MYBPC3*	NM_000256.3(MYBPC3):c.1624 + 4 A > T	rs397515916	2	Likely pathogenic	0.00001974
* MYBPC3*	NM_000256.3(MYBPC3):c.1624G > C, p.(Glu542Gln)	rs121909374	3	Pathogenic	0.00004182
* MYBPC3*	NM_000256.3(MYBPC3):c.927-9 G > A	rs397516083	1	Pathogenic	0.00001046
* MYBPC3*	NM_000256.3(MYBPC3):c.833delG, p.(Gly278GlufsTer22)	rs727503212	1	Pathogenic	0.00001258
* MYBPC3*	NM_000256.3(MYBPC3):c.772 G > A (p.Glu258Lys)	rs397516074	1	Pathogenic	0.00003216
* MYBPC3*	NM_000256.3(MYBPC3):c.26-2 A > G	rs376395543	1	Likely pathogenic	0.00005184
* MYH7*	NM_000257.4(MYH7):c.5655 G > A, p.(Ala1885 = )	rs753392652	2	Pathogenic	0.00002637
* MYH7*	NM_000257.4(MYH7):c.3133 C > T, p.(Arg1045Cys)	rs45611033	1	Likely pathogenic	0.00003871
* MYH7*	NM_000257.4(MYH7):c.2722 C > G, p.(Leu908Val)	rs121913631	1	Pathogenic	0.00006482
* MYH7*	NM_000257.4(MYH7):c.2347 C > T, p.(Arg783Cys)	rs727503258	1	Likely pathogenic	0.000008791
* MYH7*	NM_000257.4(MYH7):c.2167 C > T, p.(Arg723Cys)	rs121913630	1	Pathogenic	0.00001762
* TNNI3*	NM_000363.4(TNNI3):c.586 G > A, p.(Asp196Asn)	rs104894727	1	Likely pathogenic	0.00001554
* TNNT2*	NM_000364.4(TNNT2):c.452 G > A, p.(Arg151Gln)	rs730881101	1	Likely pathogenic	0.000008824
* GLA*	NM_000169.2(GLA):c.335 G > A, p.(Arg112His)	rs372966991	1	Pathogenic	0.00002443
Dilated cardiomyopathy (*TTN-truncating, LMNA, RBM20, PLN, BAG3, FLNC*), *N* = 29 carriers					
* TTN*	NM_001267550.2(TTN):c.107578 C > T, p.(Gln35860Ter)	rs1009131948	1	Likely deleterious	0.00002338
* TTN*	NM_001267550.2(TTN):c.107377 + 1 G > A	rs112188483	2	Likely deleterious	0.000008873
* TTN*	NM_001267550.2(TTN):c.100825 C > T, p.(Arg33609Ter)	rs1057518195	1	Likely deleterious	0
* TTN*	NM_001267550.2(TTN):c.100026_100030delAAGAC, p.(Ser33344HisfsTer7)	rs727503537	1	Deleterious	0
* TTN*	NM_001267550.2(TTN):c.97129_97130dupTA, p.(Leu32379HisfsTer4)	rs748382770	1	Deleterious	0.000008885
* TTN*	NM_001267550.2(TTN):c.95008 C > T, p.(Arg31670Ter)	rs1322596650	1	Deleterious	0.000008882
* TTN*	NM_001267550.2(TTN):c.91920 G > A, p.(Trp30640Ter)	NA	1	Deleterious	0
* TTN*	NM_001267550.2(TTN):c.91669 C > T, p.(Arg30557Ter)	NA	1	Deleterious	0
* TTN*	NM_001267550.2(TTN):c.88837 A > T, p.(Lys29613Ter)	NA	1	Deleterious	0
* TTN*	NM_001267550.2(TTN):c.85762 G > T, p.(Glu28588Ter)	NA	1	Deleterious	0
* TTN*	NM_001267550.2(TTN):c.82240 C > T, p.(Arg27414Ter)	rs766840243	1	Deleterious	0.000008892
* TTN*	NM_001267550.2(TTN):c.75865 G > T, p.(Glu25289Ter)	NA	1	Deleterious	0
* TTN*	NM_001267550.2(TTN):c.74338 C > T, p.(Arg24780Ter)	rs794729285	1	Deleterious	0.000008917
* TTN*	NM_001267550.2(TTN):c.67751 G > A, p.(Trp22584Ter)	NA	1	Deleterious	0
* TTN*	NM_001267550.2(TTN):c.67519 C > T, p.(Gln22507Ter)	NA	1	Deleterious	0
* TTN*	NM_001267550.2(TTN):c.52473 G > A, p.(Trp17491Ter)	NA	1	Deleterious	0
* TTN*	NM_001267550.2(TTN):c.52405 + 2 T > C	NA	1	Deleterious	0
* TTN*	NM_001267550.2(TTN):c.51667 C > T, p.(Arg17223Ter)	rs748956593	1	Deleterious	0
* TTN*	NM_001267550.2(TTN):c.51459_51462delTACA, p.(Asp17153GlufsTer11)	rs886043718	1	Deleterious	0
* TTN*	NM_001267550.2(TTN):c.41835 T > G, p.(Tyr13945Ter)	NA	1	Deleterious	0
* TTN*	NM_001267550.2(TTN):c.40558 + 1 G > A	rs368219776	3	Deleterious	0.0001261
* TTN*	NM_001267550.2(TTN):c.10303 + 2 T > C	rs371596417	1	Likely deleterious	0.00002642
* TTN*	NM_001267550.2(TTN):c.9571 C > T, p.(Gln3191Ter)	NA	1	Likely deleterious	0
* LMNA*	NM_170707.3(LMNA):c.1580 G > A, p.(Arg527His)	rs57520892	1	Pathogenic	0.00001762
* PLN*	NM_002667.4(PLN):c.40_42delAGA, p.(Arg14del)	rs397516784	1	Pathogenic	0.00001552
* BAG3*	NM_004281.3(BAG3):c.1353 C > G, p.(Tyr451Ter)	NA	1	Likely pathogenic	0
Arrhythmogenic right-ventricular cardiomyopathy (*PKP2, DSP, DSC2, DSG2, TMEM43*), *N* = 22 carriers					
* PKP2*	NM_004572.3(PKP2):c.2312_2313delTC, p.(Leu771ProfsTer2)	rs1064794350	1	Likely pathogenic	0
* PKP2*	NM_004572.3(PKP2):c.2146-1 G > C	rs193922674	7	Pathogenic	0.00006974
* PKP2*	NM_004572.3(PKP2):c.1162 C > T, p.(Arg388Trp)	rs766209297	2	Likely pathogenic	0
* PKP2*	NM_004572.3(PKP2):c.358 G > T, p.(Glu120Ter)	NA	1	Likely pathogenic	0
* PKP2*	NM_004572.3(PKP2):c.253_256delGAGT, p.(Glu85MetfsTer26)	rs786204388	1	Pathogenic	0.000008794
* DSP*	NM_004415.4(DSP):c.939 + 1 G > A	rs727504443	1	Pathogenic	0
* DSP*	NM_004415.4(DSP):c.3507 C > A, p.(Tyr1169Ter)	rs148894066	1	Likely pathogenic	0
* DSP*	NM_004415.4(DSP):c.4198 C > T, p.(Arg1400Ter)	rs770873593	1	Pathogenic	0
* DSC2*	NM_024422.6(DSC2):c.943-1 G > A	rs796756333	1	Pathogenic	0
* DSC2*	NM_024422.6(DSC2):c.631-2 A > G	rs397514042	2	Pathogenic	0.00003523
* DSC2*	NM_024422.6(DSC2):c.136 G > T, p.(Glu46Ter)	NA	1	Likely pathogenic	0
* DSG2*	NM_001943.5(DSG2):c.1377 T > A, p.(Tyr459Ter)	NA	1	Likely pathogenic	0
* DSG2*	NM_001943.5(DSG2):c.1773_1774delTG, p.(Cys591Ter)	rs397516703	1	Pathogenic	0.000008856
* DSG2*	NM_001943.5(DSG2):c.3059_3062delAGAG, p.(Glu1020AlafsTer18)	rs397516706	1	Likely pathogenic	0.00006186
Catecholaminergic polymorphic ventricular tachycardia (*RYR2*), *N* = 4 carriers					
* RYR2*	NM_001035.3(RYR2):c.1259 G > A, p.(Arg420Gln)	rs794728721	1	Pathogenic	0
* RYR2*	NM_001035.3(RYR2):c.1477-1 G > A	NA	1	Likely pathogenic	0
* RYR2*	NM_001035.3(RYR2):c.6555 + 1 G > A	NA	1	Likely pathogenic	0
* RYR2*	NM_001035.3(RYR2):c.9004 G > T, p.(Glu3002Ter)	NA	1	Likely pathogenic	0
Marfan syndrome and familial thoracic aortic aneurysms and dissections (*FBN1*), *N* = 1 carrier					
* FBN1*	NM_000138.4(FBN1):c.3712 G > A, p.(Asp1238Asn)	rs794728208	1	Likely pathogenic	0
Familial long-QT syndrome types 1,2, and 3 (*KCNQ1, KCNH2, SCN5A*), N = 39 carriers					
* KCNQ1*	NM_000218.3(KCNQ1):c.477 + 5 G > A	rs397508111	1	Pathogenic	0.00001758
* KCNQ1*	NM_000218.3(KCNQ1):c.502 G > A, p.(Gly168Arg)	rs179489	1	Pathogenic	0.00001772
* KCNQ1*	NM_000218.3(KCNQ1):c.604 G > A, p.(Asp202Asn)	rs199472702	1	Pathogenic/Likely pathogenic	0.000007789
* KCNQ1*	NM_000218.3(KCNQ1):c.683 + 5 G > A	rs397508122	1	Pathogenic	0.00002364
* KCNQ1*	NM_000218.3(KCNQ1):c.830 C > T p.(Ser277Leu)	rs199472730	1	Pathogenic	0
* KCNQ1*	NM_000218.3(KCNQ1):c.944 A > G, p.(Tyr315Cys)	rs74462309	1	Pathogenic	0
* KCNQ1*	NM_000218.3(KCNQ1):c.1022 C > A, p.(Ala341Glu)	rs12720459	1	Pathogenic	0
* KCNQ1*	NM_000218.3(KCNQ1):c.1085 A > G, p.(Lys362Arg)	rs12720458	3	Likely pathogenic	0.00007918
* KCNQ1*	NM_000218.3(KCNQ1):c.1096 C > T (p.Arg366Trp)	rs199473411	1	Pathogenic	0
* KCNQ1*	NM_000218.3(KCNQ1):c.1190 G > A, p.(Arg397Gln)	rs374090960	1	Likely pathogenic	0.00002639
* KCNQ1*	NM_000218.3(KCNQ1):c.1394-1 G > T	rs775537394	1	Pathogenic	0.000008795
* KCNQ1*	NM_000218.3(KCNQ1):c.1552 C > T, p.(Arg518Ter)	rs17215500	7	Pathogenic	0.0001759
* KCNQ1*	NM_000218.3(KCNQ1):c.1590 + 1 G > A	rs767921776	1	Pathogenic	0
* KCNQ1*	NM_000218.3(KCNQ1):c.1781G > A, p.(Arg594Gln)	rs199472815	1	Pathogenic	0.00004652
* KCNH2*	NM_000238.4(KCNH2):c.2350 C > T, p.(Arg784Trp)	rs12720441	3	Likely pathogenic	0.00002329
* KCNH2*	NM_172057.2(KCNH2):c.23 C > T, p.(Ala8Val)	rs972201049	2	Likely pathogenic	0.00001763
* KCNH2*	NM_000238.4(KCNH2):c.1128 G > A, p.(Gln376 = )	rs770047651	1	Likely pathogenic	0
* SCN5A*	NM_198056.2(SCN5A):c.5825_5826delCT, p.(Pro1942ArgfsTer12)	rs1553692416	3	Likely pathogenic	0
* SCN5A*	NM_198056.2(SCN5A):c.4859 C > T, p.(Thr1620Met)	rs199473282	1	Pathogenic	0.000008817
* SCN5A*	NM_198056.2(SCN5A):c.4501 C > G, p.(Leu1501Val)	rs199473266	3	Likely pathogenic	0.00004396
* SCN5A*	NM_198056.2(SCN5A):c.4132 G > A, p.(Val1378Met)	rs748312802	1	Likely pathogenic	0
* SCN5A*	NM_198056.2(SCN5A):c.3956 G > T, p.(Gly1319Val)	rs199473220	1	Likely pathogenic	0.00007282
* SCN5A*	NM_198056.2(SCN5A):c.1936delC, p.(Gln646ArgfsTer5)	rs727505158	1	Pathogenic	0.00006488
* SCN5A*	NM_198056.2(SCN5A):c.1603C > T, p.(Arg535Ter)	rs1417036453	1	Pathogenic	0

Variants with pathogenic or likely pathogenic ClinVar annotation and/or high-confidence predicted loss-of-function in coding regions were curated following ACMG/AMP Standards and Guidelines for the Interpretation of Sequence Variants, reviewed by two or more laboratory scientists and a clinical geneticist, based on human genome build GRCh37. *TTN*-truncating variants were classified as deleterious or likely deleterious using modified clinical criteria. Analysis was restricted to single nucleotide variants and small insertions/deletions. Variants of uncertain significance were excluded. For detailed variant information, including genomic coordinates, clinical annotations, supporting evidence of pathogenicity and population allele frequencies from ethnically diverse reference populations, see Supplementary Table 1.

*MAF* minor allele frequency (from the Genome Aggregation Database [gnomAD] v2.1.1), *NFE* non-Finnish European.

Variants were found in genes associated with hypertrophic cardiomyopathy (*MYBPC3, MYH7, TNNI3, TNNT2, GLA*, *N* = 24 carriers), dilated cardiomyopathy (*TTN, LMNA, PLN, BAG3*, *N* = 29 carriers), arrhythmogenic right-ventricular cardiomyopathy (*PKP2, DSP, DSC2*, *N* = 22 carriers), catecholaminergic polymorphic ventricular tachycardia (*RYR2*, *N* = 4 carriers), Marfan syndrome and familial thoracic aortic aneurysms and dissections (*FBN1*, *N* = 1 carrier), and familial long-QT syndrome types 1,2, and 3 (*KCNQ1, KCNH2, SCN5A*, *N* = 39 carriers).

*TTN*-truncating variants (TTNtvs) with high PSI were found in 25/13,131 ASPREE participants, at a prevalence of 0.2%. This was lower than rates reported from population-based studies, ranging between 0.6 and 1.2%^13^. Variants in genes associated with arrhythmogenic right-ventricular cardiomyopathy (*PKP2, DSP, DSC2, DSG2, TMEM43*) were found in 22/13,131 ASPREE participants, at a prevalence of 0.17%, also lower than rates reported in population-based studies (0.23%)^9^.

### Association of variants with presumed sudden cardiac deaths

Among the 119 variant carriers identified, we assessed presumed sudden cardiac death (SCD) or rapid cardiac death (RCD) over an average of 4.7 years of follow-up per participant. During this follow-up period, there were two related clinical adjudicated deaths noted among the variant carriers (1.7% of carriers); one coded as s*udden cardiac death* (*SCN5A*, NM_198056.2:c.4501 C > G, p.(Leu1501Val), male, age 76 years) and one as *rapid cardiac death* (*KCNQ1*, NM_000218.3:c.1781G > A, p.(Arg594Gln), female, age 85 years). The pathogenic variants identified in these participants were both found in established long-QT syndrome genes (*SCN5A* and *KCNQ1*)^5^. The rate of presumed SCD in variant carriers (1.7%, 2/119) was higher than in non-carriers (0.08%, 11/12936) (*p* < 0.001).

Among the remaining 98% (117/119) of variant carriers, there were no presumed sudden cardiac deaths during follow-up, to average age 79.3 years. Seven variant carriers died from other causes during follow-up. The remaining 110 variant carriers (92%) remained alive and free of diagnosed cardiovascular events for the follow-up period.

### Family history of cardiovascular disease

Among the 119 carriers of pathogenic/likely pathogenic or deleterious variants identified in ASPREE, 56 (47%) reported a family history of heart attack (of any kind) occurring in either in father, mother, or sibling, at the time of study enrolment. Of these, five variant carriers (4.2%) reported a family history of early-onset heart attack occurring in a family member (<50 years of age). However, these events cannot be attributed to genetic cardiovascular disorders specifically, and some may have related to coronary artery disease.

Of the two ASPREE participants with pathogenic variants in long-QT syndrome genes who died from presumed sudden or rapid cardiac death during the ASPREE trial, one had reported a family history of heart attack at enrolment (father, event at age >50 years).

## Discussion

In this study, we detected 119 individuals aged 70 years and older carrying (likely) pathogenic or deleterious variants in genes associated with inherited cardiovascular disorders, some of which are associated with sudden cardiac death. This corresponded to a carrier rate of 1 in 110 asymptomatic older adults for the 25 genes analysed. During the follow-up period, two variant carriers (1.7%) died from clinically adjudicated presumed sudden/rapid cardiac deaths, both with pathogenic variants in long-QT syndrome genes. Although this death rate was higher than in non-carriers (0.08%, *p* < 0011), the majority of variant carriers identified (98%) had no cardiovascular events occur during follow-up, at average age 79.3 years. Our study therefore represents a unique insight into the prevalence and clinical impact of pathogenic variants for inherited cardiovascular disorder genes in asymptomatic older adults, a segment of the population not typically offered genetic testing. Our study contributes to overcoming the historic clinical ascertainment bias in genotype:phenotype correlations studies of inherited cardiovascular disorders.

Genetic variants of clinical significance were detected mostly in asymptomatic older individuals without a history of cardiovascular disease. We detected a total of 89 different variants that met our inclusion criteria, 13 of which (to our knowledge) are not previously reported in the literature, with no ClinVar annotation or rsID. The identification of a pathogenic variant in the cardiac genes analysed in this study does not predict the penetrance, age of onset, or severity and course of disease. However, the same variants, when detected at younger ages in the course of clinical genetic testing, can influence clinical decisions regarding the use of interventions to prevent and manage inherited cardiovascular disorders. When decisions around such interventions are influenced by genetic testing results, the results should be supported by the most accurate and robust gene penetrance and population risk estimates possible. Unfortunately, most genes currently included on sudden cardiac death testing panels are not supported by robust gene-disease association or accurate gene penetrance estimates, complicating the use of genetic testing for the prevention and clinical management of these disorders^4,6^. Further studies are required in population-based cohorts with extended follow-up, to better understand genotype:phenotype relationships for these genes.

Overall, the number of presumed SCDs attributable to pathogenic or likely pathogenic variants detected in the ASPREE population was relatively low (2/119 or 1.7% of carriers). This reflects the reduced penetrance and variable expressivity of clinical manifestations of the inherited cardiovascular disorder genes analysed. However, although the variants were mostly found in asymptomatic individuals, the same variants could play causative roles in cardiac phenotypes or even SCDs in other individuals, including the family members of the participants identified. This reflects the paradox of inherited cardiovascular disorder genes, which have low population penetrance but severe potential clinical consequences.

The low clinical effect of the variants detected in the ASPREE population is further influenced by the unusual ascertainment of the population, comprised only of older asymptomatic individuals without a history of cardiovascular disease events. Due to the strict ASPREE trial enrolment criteria, individuals with any past history of cardiovascular events who were affected by pathogenic alleles for the genes analysed were selected out of the study population. This has created a unique ascertainment bias towards unaffected variant carriers in the ASPREE study (the opposite of historic ascertainment bias towards affected carriers, usually seen in human clinical genetic studies).

Despite the healthy selection bias in the clinical trial enrolment, two variant carriers in the ASPREE population had presumed SCDs occur during the trial period; both with pathogenic variants in long-QT syndrome genes (*SCN5A* and *KCNQ1*). These deaths indicate that the clinical risk conferred by pathogenic variants in the genes analysed still persists past the age of 70 years, and even in a highly selected population of asymptomatic adults during a relatively short follow-up period (average 4.7 years of follow-up per participant). The risk of SCD in variant carriers in the study (1.7%) was significantly higher than in non-carriers (0.08%, *p* < 0.001), indicating that these variants are still of clinical interest, even despite their reduced penetrance, due to the severity of the related outcome.

The detection of such variants in our study raises the ethical question of whether the identified genetic research findings in genes associated with inherited cardiovascular disorders should be returned to participants^22^. This could potentially mitigate future events and prompt cascade testing of younger family members. However, decisions regarding the return of the variants detected in this study are complicated, especially when the majority of variant carriers (117/119 or 98%) are asymptomatic and have survived to average age 79.3 years. In addition, we observed that several variants detected in our study were also reported in older individuals in the gnomAD reference database, who were aged 70 years and older (13 of the 89 variants detected, or 14.6%).

The low number of sudden or rapid cardiac deaths observed among variant carriers in ASPREE raises another question of whether the risk conferred by the variants may have been modified by other factors. Carriers of a given cardiac pathogenic variant within a family can potentially have a range of clinical manifestations—from asymptomatic, to asymptomatic with abnormal cardiac function, to symptomatic with clinical presentation, to sudden cardiac death. Such differences could be due to variations in a range of factors, including a background of genetic modifiers as well as epigenetic and lifestyle factors. The variable expressivity of the pathogenic variants detected in our study could be influenced by the absence of exacerbating factors that increase risk, but may also relate to an enrichment of protective factors that reduce risk. For example, protective genetic and environmental factors may have modified the risk conferred by the pathogenic variants detected—perhaps over and above the properties of the genetic variants themselves. There is increasing evidence that factors beyond just a single gene variant can modify risk of Mendelian cardiovascular conditions, including polygenic risk modification in long-QT syndrome^23^ and non-genetic factors such as exercise in arrhythmogenic right-ventricular cardiomyopathy^24^. The variant carriers identified in the present study, who remained asymptomatic to age 70 years and older, may be more likely to have benefited from a favourable lifestyle, and/or carry protective alleles may modify disease risk.

Strengths of our study include the well-characterized apparently healthy cohort, enrolled in randomized-controlled trial with a large sample size, high coverage sequencing and access to robustly ascertained and adjudicated clinical events^17^. Participants received a clinical assessment at baseline, to confirm the absence of prior cardiovascular events, dementia and physical disability to determine their eligibility. Adjudicated endpoints from the ASPREE trial then enabled measurement of incident SCDs during follow-up. Further strengths of the study include the analysis of only genes with established disease association, and application of a high standard of variant curation to reduce false positives, employing a conservative approach to variant detection.

Limitations of our study include the inability to generalise results to the broader population given the highly selected nature and predominantly European ancestry of the ASPREE cohort. A further limitation is the absence of disease-specific clinical phenotyping for inherited cardiovascular conditions; including the absence of echocardiograms and electrocardiograms which were not collected routinely at baseline as part of the ASPREE protocol, to determine the presence or absence of arrhythmogenic substrates. Therefore, our analysis was limited to hard clinical events (deaths), rather than other related phenotypes. This has likely underestimated the true clinical effect and underlying rate of cardiovascular abnormalities in the variant carriers detected. The follow-up period was also relatively short, an average of 4.7 years per participant. Our analysis of family history in variant carriers was limited to a single self-reported question on family history of heart attack of any kind. Specific questions related to a family history of inherited cardiovascular conditions (e.g. cardiomyopathy, sudden death etc) were not asked.

As genomic testing becomes more widespread, there will be an increasing propensity to identify pathogenic variants in asymptomatic adults, often in the absence of symptoms or family history of related disease. How this challenge will be handled clinically remains to be seen, especially for conditions where associated genes have low penetrance and variable expressivity. Currently, penetrance and risk estimates for many genes associated with inherited cardiovascular disorders suffer from a lack of robust population-level data. More robust data are required, especially from population-based studies with longer periods of prospective follow-up, to estimate the penetrance of these genes and thus improve the clinical utility of genetic testing as a tool in the management of inherited cardiovascular conditions.

## Methods

### Study population

Participants were enrolled in the ASPirin in Reducing Events in the Elderly (ASPREE) study, a randomized, placebo-controlled trial of daily low-dose aspirin. Study design^25^, recruitment^26^, baseline characteristics^27^, and outcomes^16–18^ have been published previously. Genetic analysis was conducted on genomic DNA samples provided by a total of 13,131 Australian participants, who were mostly of European descent (self-reported), aged 70 years or older at enrolment, with no prior cardiovascular disease events, dementia diagnosis, persistent physical disability, or serious illness likely to cause death within 5 years. Biospecimens from US ASPREE participants were not available for analysis at the time of the study. All ASPREE participants had no history of diagnosed myocardial infarction, heart failure, stroke, transient ischemic attack, atrial fibrillation, or systolic blood pressure greater than 180 mmHg at enrolment. The study is registered on Clinicaltrials.gov (NCT01038583) and was approved by the Alfred Hospital Human Research Ethics Committee.

### DNA sequencing and variant analysis

A targeted panel of 762 genes was designed^19^, including genes associated with cardiovascular diseases. Of these genes, we selected 25 genes on the basis of their routine use in genetic testing of inherited cardiovascular disorders that can cause sudden cardiac death^1,2^. We only selected genes with strong or definitive evidence of gene-disease association according to the Clinical Genome Resource (ClinGen) framework^28^. This included genes associated with hypertrophic cardiomyopathy or genocopy Fabry disease (*MYBPC3, MYH7, TNNI3, TNNT2, TPM1, ACTC1, MYL2, MYL3, GLA*), dilated cardiomyopathy (*TTN-truncating variants, LMNA, RBM20, PLN, BAG3, FLNC, DSP*), arrhythmogenic right-ventricular cardiomyopathy (*PKP2, DSP, DSC2, DSG2, TMEM43*), catecholaminergic polymorphic ventricular tachycardia (*RYR2*), Marfan syndrome and familial thoracic aortic aneurysms and dissections (*FBN1*), and familial long-QT syndrome types 1, 2, and 3 (*KCNQ1, KCNH2, SCN5A*).

Following standard protocols, DNA was extracted and sequenced using the Thermo Fisher Scientific S5TM XL system, to average 200× depth, with reads aligned to GRCh37. Variant calling followed published protocols^19^ and all variants detected were heterozygous. Variants passed the following quality control thresholds; minimum quality score >30, minimum read coverage >50 reads, balanced reference/alternative strand read coverage >30% on both strands, no homopolymers adjacent to the variant site. Variants with pathogenic or likely pathogenic annotation^29^ and/or high-confidence predicted loss-of-function in coding regions^30^ were curated following ACMG/AMP Standards and Guidelines for the Interpretation of Sequence Variants^31^, reviewed by two or more laboratory scientists and a clinical geneticist. *MYH7* variants were curated using an adapted ACMG/AMP framework^32^. *TTN*-truncating variants were classified as deleterious or likely deleterious using modified criteria^33,34^, with only variants with high exon percentages spliced in (PSI) included^35^. Analysis was restricted to single nucleotide variants and small insertions/deletions. Variants of uncertain significance were excluded.

### Sudden cardiac deaths

We assessed the outcome of sudden cardiac death (SCD) in ASPREE participants during mean 4.7 years follow-up, using adjudicated trial endpoints coded as either sudden cardiac death (SCD) or rapid cardiac death (RCD). Assessments were based on death certificates and other clinical evidence used to determine cause of death, rather than autopsy confirmation, meaning the study outcome should be considered ‘presumed SCD’. Deaths coded as SCD were defined as occurring within one hour of the onset of new cardiac symptoms (ischemic chest symptoms or sudden collapse) or unwitnessed death after last being seen without new cardiac symptoms, and in each case, without any coronary disease (clinically or at autopsy) that could be rapidly fatal. Deaths coded as RCDs were defined as deaths occurring within 1–24 h of the onset of severe cardiac symptoms unrelated to other known causes, or death in hospital after possible myocardial infarction. All deaths in the ASPREE trial were adjudicated by two reviewers with discussion to achieve consensus as needed. Coding and adjudication for ASPREE study endpoints has been described previously^16–18^.

We compared the risk of presumed SCD/RCD in ASPREE variant carriers versus non-carriers using linear regression. Non-carriers were defined as ASPREE participants where no pathogenic variant for any medically actionable gene was detected^19^. We examined family history of heart attack in variant carriers based on self-reported questionnaires administered at the time of study enrolment (response = yes for heart attack in father, mother, or sibling).

### Ethics statement

This work was approved by the Alfred Hospital Human Research Ethics Committee (Project 390/15) in accordance with the National Statement on Ethical Conduct in Human Research (2007). Informed consent for genetic analysis was obtained from all study participants.

### Reporting summary

Further information on research design is available in the Nature Research Reporting Summary linked to this article.

## Supplementary information

Supplementary Data 1

Reporting Summary

## Data Availability

Sequence and phenotype data that support the findings of this study have been deposited in the European Genome-phenome Archive (EGAS00001005316).

## Code availability

The software code used for the analysis of the current study is available from the corresponding author on reasonable request.
