# Supplementary material for: Genetic variants associated with inherited cardiovascular disorders among 13,131 asymptomatic older adults of European descent
Source: NPJ Genom Med. 2021 Jun 16;6:51. doi: 10.1038/s41525-021-00211-x (PMC8209162; doi:10.1038/s41525-021-00211-x)
Supplement: Supplementary file 2 — Reporting Summary [file 41525_2021_211_MOESM2_ESM.pdf]

nature research

Corresponding author(s): PAUL LACAZE
Last updated by author(s): May 20, 2021

Reporting Summary

Nature Research wishes to improve the reproducibility of the work that we publish. This form provides structure for consistency and transparency in reporting. For further information on Nature Research policies, see our Editorial Policies and the Editorial Policy Checklist.

Statistics

For all statistical analyses, confirm that the following items are present in the figure legend, table legend, main text, or Methods section.

n/a	Confirmed	
☐	☒	The exact sample size (n) for each experimental group/condition, given as a discrete number and unit of measurement
☐	☒	A statement on whether measurements were taken from distinct samples or whether the same sample was measured repeatedly
☐	☒	The statistical test(s) used AND whether they are one- or two-sided<br>*Only common tests should be described solely by name; describe more complex techniques in the Methods section.*
☐	☒	A description of all covariates tested
☐	☒	A description of any assumptions or corrections, such as tests of normality and adjustment for multiple comparisons
☒	☐	A full description of the statistical parameters including central tendency (e.g. means) or other basic estimates (e.g. regression coefficient) AND variation (e.g. standard deviation) or associated estimates of uncertainty (e.g. confidence intervals)
☒	☐	For null hypothesis testing, the test statistic (e.g. F, t, r) with confidence intervals, effect sizes, degrees of freedom and P value noted<br>*Give P values as exact values whenever suitable.*
☒	☐	For Bayesian analysis, information on the choice of priors and Markov chain Monte Carlo settings
☒	☐	For hierarchical and complex designs, identification of the appropriate level for tests and full reporting of outcomes
☒	☐	Estimates of effect sizes (e.g. Cohen's d, Pearson's r), indicating how they were calculated

Our web collection on statistics for biologists contains articles on many of the points above.

Software and code

Policy information about availability of computer code

Data collection: Data analysis was conducted using R.

Data analysis: Data analysis was conducted using R.

For manuscripts utilizing custom algorithms or software that are central to the research but not yet described in published literature, software must be made available to editors and reviewers. We strongly encourage code deposition in a community repository (e.g. GitHub). See the Nature Research guidelines for submitting code & software for further information.

Data

Policy information about availability of data

All manuscripts must include a data availability statement. This statement should provide the following information, where applicable:

- Accession codes, unique identifiers, or web links for publicly available datasets
- A list of figures that have associated raw data
- A description of any restrictions on data availability

Sequence and phenotype data that support the findings of this study have been deposited in the European Genome-phenome Archive (EGAS00001005316).

Field-specific reporting

Please select the one below that is the best fit for your research. If you are not sure, read the appropriate sections before making your selection.

☒ Life sciences ☐ Behavioural & social sciences ☐ Ecological, evolutionary & environmental sciences

For a reference copy of the document with all sections, see nature.com/documents/nr-reporting-summary-flat.pdf

Life sciences study design

All studies must disclose on these points even when the disclosure is negative.

Sample size	13,131
Data exclusions	None
Replication	n/a
Randomization	n/a
Blinding	n/a

Reporting for specific materials, systems and methods

We require information from authors about some types of materials, experimental systems and methods used in many studies. Here, indicate whether each material, system or method listed is relevant to your study. If you are not sure if a list item applies to your research, read the appropriate section before selecting a response.

Materials & experimental systems

n/a	Involved in the study	
☒	☐	Antibodies
☒	☐	Eukaryotic cell lines
☒	☐	Palaeontology and archaeology
☒	☐	Animals and other organisms
☐	☒	Human research participants
☐	☒	Clinical data
☒	☐	Dual use research of concern

Methods

n/a	Involved in the study	
☒	☐	ChIP-seq
☒	☐	Flow cytometry
☒	☐	MRI-based neuroimaging

Human research participants

Policy information about studies involving human research participants

Population characteristics	Participants were enrolled in the ASPirin in Reducing Events in the Elderly (ASPREE) study, a randomized, placebo-controlled trial of daily low-dose aspirin. Genetic analysis was conducted on genomic DNA samples provided by a total of 13,131 Australian participants, who were mostly of European descent (self-reported), aged 70 years or older at enrolment, with no prior cardiovascular disease events, dementia diagnosis, persistent physical disability, or serious illness likely to cause death within 5 years.
Recruitment	Participants were recruited from the community through general practitioners
Ethics oversight	The study was approved by the Alfred Hospital Human Research Ethics Committee.

Note that full information on the approval of the study protocol must also be provided in the manuscript.

Clinical data

Policy information about clinical studies

All manuscripts should comply with the ICMJE guidelines for publication of clinical research and a completed CONSORT checklist must be included with all submissions.

Clinical trial registration	NCT01038583
Study protocol	https://aspree.org/aus/wp-content/uploads/sites/2/2014/04/ASPREE-Protocol-AUS-Version-9-Nov-2014.pdf
Data collection	https://aspree.org/aus/wp-content/uploads/sites/2/2014/04/ASPREE-Protocol-AUS-Version-9-Nov-2014.pdf

Outcomes

We assessed the outcome of sudden cardiac death (SCD) in ASPREE participants during mean 4.7 years follow-up, using adjudicated trial endpoints coded as either sudden cardiac death (SCD) or rapid cardiac death (RCD). Assessments were based on death certificates and other clinical evidence used to determine cause of death, rather than autopsy confirmation, meaning the study outcome should be considered 'presumed SCD'. Deaths coded as SCD were defined as occurring within one hour of the onset of new cardiac symptoms (ischemic chest symptoms or sudden collapse) or unwitnessed death after last being seen without new cardiac symptoms, and in each case, without any coronary disease (clinically or at autopsy) that could be rapidly fatal. Deaths coded as RCDs were defined as deaths occurring within 1-24 hours of the onset of severe cardiac symptoms unrelated to other known causes, or death in hospital after possible myocardial infarction. All deaths in the ASPREE trial were adjudicated by two reviewers with discussion to achieve consensus as needed.
